# Short-Form Video Exposure and Its Two-Sided Effect on the Physical Activity of Older Community Women in China: Secondary Data Analysis

**DOI:** 10.2196/45091

**Published:** 2023-09-13

**Authors:** Chen Wu, Si Chen, Shan Wang, Sijing Peng, Jiepin Cao

**Affiliations:** 1 School of Nursing and Rehabilitation, Cheeloo College of Medicine, Shandong University, Jinan, China; 2 Department of Population Health, Grossman School of Medicine, New York University, New York, NY, United States

**Keywords:** short-form video, media exposure, physical activity, step counts, older adults, apps

## Abstract

**Background:**

There is a tendency for older adults to become more physically inactive, especially older women. Physical inactivity has been exacerbated since the COVID-19 pandemic. Lockdowns and information-based preventive measures for COVID-19 increased the number of short-form video app users and short-form video exposure, including content exposure and the duration of exposure, which has demonstrated important effects on youths’ health and health-related behaviors. Despite more older adults viewing short-form videos, less is known about the status of their short-form video exposure or the impacts of the exposure on their physical activity.

**Objective:**

This study aims to describe physical activity–related content exposure among older adults and to quantify its impacts along with the duration of short-form video exposure on step counts, low-intensity physical activity (LPA), and moderate-to-vigorous physical activity (MVPA).

**Methods:**

We analyzed a subsample (N=476) of older women who used smartphones and installed short-form video apps, using the baseline data collected from an ongoing cohort study named the Physical Activity and Health in Older Women Study (PAHIOWS) launched from March to June 2021 in Yantai, Shandong Province, China. The information on short-form video exposure was collected by unstructured questions; physical activity–related content exposure was finalized by professionals using the Q-methodology, and the duration of exposure was transformed into hours per day. Step counts, LPA, and MVPA were assessed with ActiGraph wGT3X-BT accelerometers. Multiple subjective and objective covariates were assessed. Linear regression models were used to test the effects of short-form video exposure on step counts, LPA, and MVPA. MVPA was dichotomized into less than 150 minutes per week and 150 minutes or more per week, and the binary logistic regression model was run to test the effects of short-form video exposure on the achievement of spending 150 minutes or more on MVPA.

**Results:**

Of 476 older women (mean age 64.63, SD 2.90 years), 23.7% (113/476) were exposed to physical activity–related short-form videos, and their daily exposure to short-form videos was 1.5 hours. Physical activity–related content exposure increased the minutes spent on MVPA by older women (B=4.14, 95% CI 0.13-8.15); the longer duration of short-form video exposure was associated with a reduced step count (B=−322.58, 95% CI −500.24 to −144.92) and minutes engaged in LPA (B=−6.95, 95% CI −12.19 to −1.71) and MVPA (B=−1.56, 95% CI −2.82 to −0.29). Neither content exposure nor the duration of exposure significantly increased or decreased the odds of older women engaging in MVPA for 150 minutes or more per week.

**Conclusions:**

Short-form video exposure has both positive and negative impacts on the physical activity of older adults. Efforts are needed to develop strategies to leverage the benefits while avoiding the harms of short-form videos.

## Introduction

Globally, populations are aging, and physical activity (PA) is an essential parameter of healthy aging in societies worldwide, as being physically active can reduce the risk of all-cause mortality, cognition decline, functional limitation, and psychological problems of older adults [1,2]. Although the World Health Organization recommends at least 150 minutes of moderate-intensity PA per week or at least 75 minutes of vigorous-intensity PA for older adults, few of them could meet the guidelines. Most older adults engage in low-intensity PA (LPA) in their daily lives [3], and the most common type of PA is step based [4]. In recent years, studies have found that engaging in LPA provides health benefits, such as improved sleep quality [5] and reduced incidence of heart failure rehospitalization [6]. Additionally, taking more steps per day could decrease depression and all-cause mortality in later life regardless of the stepping intensity [7,8]. However, there is a tendency for older adults to become more physically inactive [9], and compared to older men, older women are generally more sedentary and less active [10,11]. This phenomenon has been exacerbated during the COVID-19 pandemic [12]. Short-form video consumption may be the reason underlying this phenomenon.

To keep entertained while staying safe indoors due to lockdowns or quarantines for COVID-19, people increased the use of digital media [13], and the short-form video exploded in popularity. Statistics reported in December 2020 showed that more than two-thirds of consumers in the United States spent between 30 minutes to 3 hours daily watching short-form videos [14]. In China, according to the Chinese Statistical Report on Internet Development by the China Internet Network Information Center, the number of short-form video users has increased by 100 million and reached 873 million from March to December 2020 [15], and individuals typically spent more than 18 hours per week watching short-form videos [16]. Older adults prefer to have in-person interactions; COVID-19 serves as a reminder of the existing “digital divide” between younger and older adults and an opportunity to bring all generations together by helping bridge digital divides [17,18]. In China, a lot of older adults who are not netizens (ie, internet users) gained access to the internet and learned skills to use smartphones with the help of their children, relatives, and community volunteers during the pandemic motivated by the lockdown and adhering to an information-based preventive measure (ie, a color-based QR code on contact-tracing apps to determine an individual’s exposure risk to COVID-19) [19,20]. The number of older netizens doubled and reached 110.8 million by December 2020 [15], and 31.3% of new older netizens were short-form video users [16]. Short-form video platforms have higher user stickiness, and short-form video exposure may continue in the daily lives of older adults beyond the types of lockdown measures from COVID-19.

Much research has been done on media exposure in youth cohorts, yet the term media exposure is not explicitly defined in the literature. Most studies in youth cohorts used content exposure or the duration of exposure as proxies of media exposure, which can guide the operationalization of the short-form video exposure concept. Social contagion theory proposes that emotions or behaviors would be rapidly spread from one individual to another, sometimes without rational thought and reason [21]. This notion is empirically supported by the content exposure literature. For example, Ngqangashe and Backer [22] found that adolescents exposed to sweet snack videos were less likely to choose fruits and vegetables, and those exposed to fruit and vegetable videos were less likely to select sweet snacks. As to the duration of exposure, the displacement hypothesis proposed that web-based communication would reduce digital media users’ psychological well-being by replacing time spent with strong ties or close relationship partners [23], and this hypothesis was corroborated by a lot of evidence. In addition to psychological impacts, increased media exposure was found to be associated with behavioral problems in youth [24]. With the rise of older netizens in the internet world, the challenges of short-form video exposure on older adults’ health also emerge. One recent study found that exposure to food videos and longer duration of exposure to short-form videos had significant effects on the overweight and obesity of older adults [25]. However, to our knowledge, less is known about PA-related short-form video exposure in older adults, and the impacts of PA-related short-form video exposure and the duration of exposure on the PA of older adults remain unclear.

To address these research gaps and advance science in older adults’ PA, especially the population less likely to engage in PA (ie, older women [10,11]), this exploratory study was designed to describe PA-related short-form video exposure among older adults and test the impacts of content exposure and the duration of exposure on their step counts, LPA, and moderate-to-vigorous PA (MVPA), respectively.

## Methods

### Participants

This secondary analysis used baseline data collected from an ongoing cohort study named the Physical Activity and Health in Older Women Study (PAHIOWS) launched from March to June 2021 in Yantai, Shandong Province, China. All investigators in PAHIOWS were trained to collect self-reported and objective data in advance by the principal investigator. Following the gatekeeper’s (ie, the director of the community center) approval, the recruitment of participants was launched. Traditional approaches including poster campaigns and person-to-person interactions were used to recruit participants. To entice participation, both monetary (gifts) and nonmonetary incentives were used. The nonmonetary incentive was a printed health report including the information on body composition assessed by Tanita MC-180 (Bailida Co, Tokyo, Japan) and arterial stiffness and vascular obstruction assessed by an automatic oscillometer device (VaSera VS-1500AE, Fukuda-Denshi, Tokyo, Japan). The inclusion criteria set for the parent study were community women ages 60-70 years who could communicate, had no cognitive impairment (the Mini-Mental State Examination score: >17 for illiteracy, >20 for primary school, and >24 for middle school and above), and provided written informed consent. There were 1370 older women enrolled in PAHIOWS; all data were collected in person, and a double-checking strategy was used to eliminate data entry mistakes. In our study, we solely used data from participants using smartphones with short-form video apps Tiktok/Douyin or Kuaishou, Toutiao, and Haokan, as they were apps older adults often used in China.

### Ethics Approval

The ethical oversight of the parent study was obtained from the institutional review board in the School of Nursing and Rehabilitation, Shandong University, China (2020-R001).

### Measurements

#### Response Variable: Physical Activity

Physical activity including step counts, LPA, and MVPA was assessed by the ActiGraph wGT3X-BT accelerometers (ActiGraph, Pensacola, FL), which is a valid tool to assess the intensity of PA [26] and step counts [27]. The location of the accelerometer may influence its validity, and wearing accelerometers on the hip is recommended by the manufacturer for free-living adults. Trained investigators used consistent instructions to guide participants to wear the accelerometers on the hip using a hip-worn belt on the spot and to wear the device all day except when sleeping, swimming, or showering. We collected all devices 7 days later, and within this time frame, two reminders were sent out by phone calls to enhance participants’ adherence to wearing the accelerometer and wearing it as instructed by investigators. As such, the random error was also reduced, and therefore, the reliability of this tool was maintained. Raw data were documented 30 times per second and were transformed into counts of movement in an epoch length of 60 seconds by ActiLife software 6.13.4 (ActiGraph). Nonwearing time was defined as an interval of at least 90 consecutive minutes of zero counts with an allowance of up to 2 minutes of 0-100 counts. A valid day is defined as a minimum wearing time of 10 hours, and the data of participants with at least 4 valid days were included for analyses, following recent recommendations [28]. Daily LPA (100-1951 counts per minute) and MVPA (1952 counts and above per minute) were classified based on the Freedson criterion [29], which is widely used in the literature and applicable to older adults, and were exported along with daily step counts.

#### Predicting Variable: Short-Form Video Exposure

Short-form video exposure was evaluated by two unstructured questions, covering content exposure (ie, what kinds of short-form videos do you often view?) and the duration of exposure per day (ie, how much time do you spend on watching short-form videos per day?). Participants’ responses to the former question constituted the Q sample; 14 professionals with expertise in kinesiology and exercise science evaluated the relevance of content with PA following the steps of the Q-methodology [30], and participants with any response rated as most relevant to PA were coded as PA-related content exposure. The duration of exposure was not PA specific, and participants’ responses to the duration of exposure were all transformed into hours per day.

#### Covariates

Sociodemographic characteristics measured included variables of age, education background (elementary school, middle and high school, or college and university), and living alone (yes or no).

The health characteristics assessed included BMI (BMI<18.5 kg/m², 18.5 kg/m² to <28 kg/m², or BMI≥28 kg/m²), history of falls (yes or no), history of falls with injuries (yes or no), history of heart diseases or otolithiasis (yes or no), balance index level, gait speed, musculoskeletal pain, and forced vital capacity.

The balance index level was tested with the Super Balance III Static Balance Test System (Acmeway, Beijing, China). Participants were instructed to complete a couple of static moves and hold each position for 30 seconds on the force platform, and the trajectory and velocity data of the center of gravity movement was documented by the system and automatically transformed into three balance index levels of low, medium, and high.

Gait speed was tested with a 5-meter walk. We measured the time walked over 5 meters at a normal pace from a moving start with a manual stopwatch and calculated the speed. Gait speed was dichotomized into two groups (<1.0 m/s and ≥1.0 m/s), with reference to the Asian Working Group for Sarcopenia consensus in 2019 [31].

We used self-reported questions to ascertain participants’ musculoskeletal pain at seven sites: knees (×2), foot (×2), spine (×1), and hips (×2). Participants reporting yes to any pain site were classified into the musculoskeletal pain group, and those without pain at any site were classified as the no musculoskeletal pain group.

Forced vital capacity was tested by a spirometer (CMCS-FHL, Beijing, China) and was measured from the end of inspiration to the end of expiration while participants were in the standing position. Forced vital capacity was dichotomized into <2500 ml and ≥2500 ml based on the clinical criterion.

### Statistical Analysis

No outlier was detected by visualizing the data with a box plot, and no strategy was used to handle the missing values in this study as the number of missing values was extremely small (around 1%). Sample characteristics were presented as means (SDs) or medians (IQRs) for continuous variables and presented as numbers (percentages) for categorical variables. The content exposure and the duration of exposure were regressed on step counts, LPA, and MVPA, respectively. Covariates including age, education background, living alone, BMI, history of falls, history of heart diseases or otolithiasis, balance index level, gait speed, musculoskeletal pain, and forced vital capacity were controlled for each regression model. BMI, education background, and the balance index level were transformed into dummy variables before entering each linear regression model. For MVPA, we did an additional analysis by dichotomizing MVPA into below 150 minutes per week and 150 minutes and above per week, and regressed content exposure and the duration of exposure on it while controlling for all covariates. We also did sensitivity analyses by replacing the history of falls with the history of falls with injuries in all models. The unstandardized regression coefficient (B), odds ratio (OR) and its 95% CI were calculated. All analyses were performed with SPSS (version 26; IBM Corp), and *P*<.05 was considered statistically significant.

## Results

### Sample Characteristics

Table 1 summarizes the characteristics of the sample. Older women in this secondary analysis had a mean age of 64.63 (SD 2.90) years, with the majority receiving education at the middle and high school level (n=351, 73.7%), living with others (n=426, 89.5%), and having abnormal BMI (n=377, 79.2%). Around one-third of participants had a history of falling (n=133, 27.9%) or a history of heart disease or otolithiasis (n=157, 33%), and 24.6% (n=117) of participants reported a history of falling with injuries. Most of the 476 participants were graded as low on the balance index level (n=383, 80.5%) and had forced vital capacity lower than 2500 ml (n=392, 82.4%). Approximately two-thirds of participants had musculoskeletal pain (n=310, 65.1%), and as many as 97.1% (n=462) of participants in this study demonstrated satisfactory gait speed (ie, ≥1 m/s). There were 23.7% (n=113) of participants exposed to PA-related short-form videos, and the median duration of exposure to short-form videos was 1.5 (IQR 1-2.5) hours per day. Participants walked an average of 8186.84 steps per day monitored by ActiGraph wGT3X-BT, and the mean (SD) time spent on LPA and MVPA was 303.15 (76.60) and 32.11 (18.83) minutes per day, respectively. Only a few participants (n=71, 14.9%) in this study spent 150 minutes or more on MVPA weekly.

**Table 1.** Sample characteristics of older women (N=476).

Variables			Values
Age (years), mean (SD)			64.63 (2.90)
**Education background, n (%)**			
	Elementary school		53 (11.1)
	Middle and high school		351 (73.7)
	College and university		72 (15.1)
**Living alone, n (%)** ^**a**^			
	Yes		48 (10.1)
	No		426 (89.5)
**BMI (kg/m²), n (%)**			
	<18.5		0 (0.0)
	18.5 to <24		99 (20.8)
	24 to <28		232 (48.7)
	≥28		145 (30.5)
**History of falling, n (%)**			
	Yes		133 (27.9)
	No		343 (72.1)
**History of falling with injuries, n (%)**			
	Yes		117 (24.6)
	No		359 (75.4)
**History of heart diseases or otolithiasis, n (%)**			
	Yes		157 (33.0)
	No		319 (67.0)
**Balance index level, n (%)**			
	Low		383 (80.5)
	Medium		78 (16.4)
	High		15 (3.2)
**Gait speed (m/s), n (%)** ^**b**^			
	<1		11 (2.3)
	≥1		462 (97.1)
**Musculoskeletal pain, n (%)**			
	Yes		310 (65.1)
	No		166 (34.9)
**Forced vital capacity (ml), n (%)**			
	<2500		392 (82.4)
	≥2500		84 (17.6)
**Short-form video exposure**			
	**Physical activity–related content exposure, n (%)**		
		Yes	113 (23.7)
		No	363 (76.3)
	The duration of exposure (hours/day), median (IQR)^c^		1.5 (1-2.5)
**Physical activity, mean (SD)**			
	Step counts (steps/day)		8186.84 (2621.88)
	Low-intensity physical activity (minutes/day)		303.15 (76.60)
	Moderate-to-vigorous physical activity (minutes/day)		32.11 (18.83)

a Missing values: n=2.

b Missing values: n=3.

c Missing value: n=1.

### Associations of Short-Form Video Exposure With the Step Counts, LPA, and MVPA of Older Women

Table 2 presents the results of linear regression models on step counts, LPA, and MVPA. When holding other variables as constant, a 1-unit increase in the duration of exposure to short-form videos significantly decreased 322.58 walk steps per day for older women. We also found significantly negative impacts of the duration of exposure to short-form videos on the minutes spent on the LPA (B=−6.95, 95% CI −12.19 to −1.71) and MVPA (B=−1.56, 95% CI −2.82 to −0.29) of older women. PA-related content exposure was only found to be significantly associated with increases in the minutes spent on MVPA (B=4.14, 95% CI 0.13-8.15). In the binary logistic regression model, the content exposure failed to significantly increase the odds of spending 150 minutes or more on MVPA (OR 1.313, 95% CI 0.723-2.381), and the duration of exposure failed to significantly reduce the odds (OR 0.949, 95% CI 0.772-1.167). The results remained stable when replacing the variable of history of fall with history of fall with injuries in each model.

**Table 2.** Factors associated with step counts, lower-intensity physical activity, and moderate-to-vigorous physical activity of community older women (N=476).

			Step counts, B (95% CI)				Low-intensity physical activity, B (95% CI)				Moderate-to-vigorous physical activity, B (95% CI)			
			Model 1	*P* value	Model 2	*P* value	Model 1	*P* value	Model 2	*P* value	Model 1	*P* value	Model 2	*P* value
Age			–50.27 (–135.61 to 35.07)	.25	–50.17 (–135.53 to 35.18)	.25	–3.70 (–6.20 to –1.19)	.004	–3.69 (–6.20 to –1.20)	.004	–0.34 (–0.94 to 0.27)	.28	–0.33 (–0.94 to 0.27)	.28
**Education background (reference: elementary school**)														
	Middle and high school		–38.62 (–811.08 to 733.83)	.92	–36.61 (–808.85 to 735.74)	.93	–16.39 (–39.15 to 6.38)	.16	–16.39 (–39.16 to 6.37)	.16	3.51 (–1.99 to 9.01)	.21	3.51 (–1.99 to 9.00)	.21
	College and university		–450.93 (–1418.28 to 516.43)	.36	–456.87 (–1424.63 to 510.89)	.35	–15.53 (–43.98 to 12.91)	.28	–15.57 (–44.03 to 12.88)	.28	–1.98 (–8.85 to 4.89)	.57	–2.03 (–8.90 to 4.84)	.56
**Living alone (reference: no**)														
	Yes		–210.57 (–993.93 to 572.79)	.60	–213.71 (–997.11 to 569.69)	.60	4.55 (–18.55 to 27.65)	.70	4.53 (–18.57 to 27.63)	.70	–3.08 (–8.66 to 2.50)	.28	–3.10 (–8.68 to 2.47)	.28
**BMI (kg/m^2^; reference: 18.5 to <24**)														
	24 to <28		99.55 (519.14 to 718.22)	.75	72.69 (–544.84 to 690.24)	.82	17.24 (–1.01 to 35.48)	.06	17.11 (–1.10 to 35.32)	.07	–2.09 (–6.50 to 2.31)	.35	–2.25 (–6.64 to 2.15)	.32
	≥28		–67.94 (–749.45 to 613.58)	.85	–90.54 (–772.51 to 591.44)	.79	27.58 (7.55 to 47.61)	.007	27.47 (7.43 to 47.51)	.007	–3.99 (–8.83 to 0.84)	.11	–4.13 (–8.97 to 0.70)	.09
**History of fall (reference: no**)														
	Yes		–564.57 (–1102.76 to –26.39)	.04	—^a^	—	–2.71 (–18.51 to 13.10)	.74	—	—	–3.39 (–7.21 to 0.43)	.08	—	—
**History of fall with injuries (reference: no**)														
	Yes		—	—	–586.44 (–1146.69 to –26.20)	.04	—	—	–2.96 (–19.39 to 13.48)	.72	—	—	–3.66 (–7.63 to 0.31)	.07
**History of heart diseases or otolithiasis (reference: yes**)														
	No		45.55 (–476.29 to 567.39)	.86	42.27 (–480.03 to 564.57)	.87	0.44 (–14.87 to 15.76)	.96	0.41 (–14.92 to 15.74)	.96	0.12 (−3.58 to 3.82)	.95	0.088 (–3.61 to 3.79)	.96
**Balance index level (reference: low**)														
	Medium		159.20 (–498.41 to 816.81)	.63	184.73 (–472.39 to 841.86)	.58	2.69 (–16.61 to 21.98)	.79	2.80 (–16.47 to 22.08)	.78	–1.77 (–6.43 to 2.89)	.46	–1.62 (–6.27 to 3.03)	.49
	High		–492.97 (–1884.22 to 898.27)	.49	–474.54 (–1865.52 to 916.44)	.50	–1.93 (–42.96 to 39.09)	.93	–1.85 (–42.87 to 39.17)	.93	–7.87 (–17.78 to 2.03)	.12	–7.77 (–17.67 to 2.13)	.12
**Gait speed (m/s) (reference: <1**)														
	≥1		1252.66 (–307.40 to 2812.71)	.12	1224.28 (–338.31 to 2786.86)	.12	–4.73 (–50.73 to 41.27)	.84	–4.91 (–50.97 to 41.16)	.83	9.76 (–1.35 to 20.86)	.09	9.55 (–1.57 to 20.67)	.09
**Musculoskeletal pain (reference: no**)														
	Yes		–441.71 (–950.59 to 67.16)	.09	–447.47 (–955.72 to 60.78)	.08	–6.70 (–21.67 to 8.27)	.38	–6.71 (–21.66 to 8.24)	.38	–2.80 (–6.41 to 0.82)	.13	–2.82 (–6.43 to 0.79)	.13
**Forced vital capacity (ml; reference: <2500**)														
	≥2500		131.63 (–490.57 to 753.83)	.68	122.54 (–500.04 to 745.12)	.70	12.78 (–5.49 to 31.04)	.17	12.73 (–5.54 to 31.00)	.17	5.29 (0.88 to 9.70)	.02	5.23 (0.82 to 9.64)	.02
**Short-form video exposure**														
	**Content exposure (reference: no**)													
		Yes	370.94 (–194.07 to 935.94)	.20	390.43 (–174.15 to 955.00)	.18	2.49 (–14.10 to 19.09)	.77	2.58 (–13.99 to 19.16)	.76	4.14 (0.13 to 8.15)	.04	4.25 (0.25 to 8.25)	.04
	**Duration of exposure (h**)		–322.58 (–500.24 to –144.92)	<.001	–322.05 (–499.76 to –144.35)	<.001	–6.95 (–12.19 to –1.71)	.01	–6.95 (–12.18 to –1.71)	.01	–1.56 (–2.82 to –0.29)	.02	–1.55 (–2.81 to –0.28)	.02
Adjusted *R*^2^			0.085	—	0.085	—	0.070	—	0.070	—	0.102	—	0.103	—

a Not applicable.

### Associations of Sample Characteristics With the Step Counts, LPA, and MVPA of Older Women

As depicted in Table 2, we found LPA reduced with advancing age (B=−3.70, 95% CI −6.20 to −1.19). A history of falling significantly reduced daily steps (B=564.57, 95% CI −1102.76 to −26.39) for older women, but failed to significantly reduce the minutes spent on LPA and MVPA. When replacing the history of fall with the history of fall with injuries, we found the history of fall with injuries significantly reduced daily steps (B=−586.44, 95% CI −1146.69 to −26.20). A BMI of 28 and above was significantly associated with increases in the minutes older women spent on LPA (B=27.58, 95% CI 7.55-47.61), and forced vital capacity of 2500 ml and above was found to be significantly associated with increases in the minutes spent on MVPA (B=5.29, 95% CI 0.88-9.70). In the binary logistic regression model, forced vital capacity of 2500 ml and above significantly increased the odds of spending 150 minutes or more on MVPA (OR 2.247, 95% CI 1.224-4.125), while a history of falling significantly reduced the odds (OR 0.423, 95% CI 0.204-0.877). Significant results remained stable when replacing the history of falls with the history of falls with injuries in each model.

## Discussion

### Principal Findings

Quarantine, limited in-person gatherings, and other lockdown measures as a result of COVID-19 have restricted PA [32,33] and simultaneously increased the number of older adult netizens [15]. Approximately one-third of new older adult netizens were short-form video users, and short-form video apps have demonstrated higher user stickiness, indicating that users would stay longer and frequently revisit [16]. Furthermore, empirical evidence from younger adults demonstrated that social media exposure influences their health behaviors such as food choice and smoking behavior [25,34]. However, less is known about the status of short-form video exposure and its association with PA among older adults. By analyzing 476 older women who were users of short-form video apps, we found that 23.7% (n=113) of them were exposed to PA-related short-form videos, and their daily exposure to short-form videos was 1.5 hours. In addition, we identified that the duration of exposure was significantly associated with reduced step counts and minutes engaged in LPA and MVPA. PA-related content exposure was significantly associated with an increase in minutes spent on MVPA but failed to significantly increase the odds of engaging in MVPA for 150 minutes or more per week among older adults.

Evidence has demonstrated that taking at least 7500 steps daily generates health benefits. For example, Saint-Maurice et al [35] analyzed data collected from 4840 adults (mean age 56.8 years; n=2435, 54% women) and found that taking 8000 steps per day was associated with significantly lower all-cause mortality compared with taking 4000 steps per day. Lee et al [7] analyzed data collected from 16,741 older women (mean age 72 years) and found that mortality rates progressively decreased before leveling at approximately 7500 steps per day. In this study, older women took an average of 8186.84 steps daily (ie, exceeding 7500 steps), and we found that a 1-hour increase in short-form video exposure significantly reduced 322.8 steps per day. This finding indicates that research is needed to explore the appropriate time spent on short-form videos by older women, and such efforts might inform strategies for app developers and community health workers to avoid the hazardous impacts of short-form videos on the health of older women.

We found that women spent an average of 303.15 minutes per day on LPA and 32.11 minutes per day on MVPA, and approximately 15% of older women would achieve the goal of spending 150 minutes or more on MVPA per week, which was slightly higher than the rates reported in studies from high-income countries [36,37]. The longer duration of exposure was significantly associated with a reduction in the minutes spent on LPA and MVPA, and the PA-related content exposure was significantly associated with an increase in the minutes spent on MVPA. Neither the duration of exposure nor PA-related content exposure was found to be significantly associated with older adults’ engagement in MVPA for 150 minutes or more per week. Being physically active is beneficial for healthy aging [1,2]; our findings strengthen the necessity of investigating the recommended time spent on short-form video apps. In addition, we found that less than one-quarter of older women viewed short-form videos related to PA. Short-form video apps often recommend content starting from the interests or preferences of the users; our findings indicate that short-form video apps may optimize the recommendation system such as passively pushing PA-related short-form videos, and such efforts may increase the content-related exposure of older women and promote engaging in more MVPA.

Except for media exposure, we found several sample characteristics that were associated with PA in older women. In this study, 27.9% (133/476) of older women reported a history of falls, which was slightly lower than those reported in older adults of both genders (ie, 30%-50%) [38], and women with a history of falls would take 564.57 fewer steps per day than those without, reducing the odds of achieving the goal of spending 150 minutes or more per week on MVPA. We also found that older women with a BMI of 28 kg/m^2^ would spend an extra 27.58 minutes per day on LPA compared with those with normal BMI, and this is inconsistent with the evidence in the literature that PA declines with an increase in weight [39]. This inconsistency can be explained by the historical effect of COVID-19 (ie, obesity is a well-known risk factor for COVID-19, severe COVID-19, and its complications, and when sparked by the pandemic, older women with a BMI above 28 kg/m^2^ may have become more sensitive to their health and formed new behaviors such as being physically active to protect or enhance their well-being [40,41]). Last, forced vital capacity declined by 20-30 ml per year starting at the age of 30 years [42], and in this study, only 17.6% (84/476) of older women had forced vital capacity of 2500 ml or more. Exercise limitation is a well-known consequence of respiratory conditions [43], our study corroborated this notion and found that compared with women with unsatisfactory forced vital capacity, women with a forced vital capacity ≥2500 ml were more likely to have spent more minutes on MVPA and to achieve the goal of engaging in MVPA for 150 minutes or more per week.

### Limitations and Future Work

This study is not free of limitations. First, limited by the secondary data analysis, we failed to assess and control variables that might influence the inference of our study. For example, older adults are accustomed to using traditional media [44], which may also transfer PA-related information and influence the outcome of interest in this study. Additionally, the built environment is particularly relevant to the PA of older adults [45,46], and this variable was not included in PAHIOWS either. Future studies may want to corroborate findings from this study by assessing and controlling these variables. Second, we failed to document the time older women spent specifically watching PA-related short-form videos. The duration of time older women spent watching PA-related short-form videos may provide a better understanding of the impact of content exposure on PA. However, collecting and calculating PA-related short-form video exposure would be challenging; future studies may want to develop strategies to collect these data and that may help give more specific recommendations for the PA of older adult netizens. Third, we found opposite conclusions on relationships between content exposure with PA and the duration of exposure with PA. Moreover, empirical evidence has demonstrated that media exposure would bring mental health benefits to older adults [47]. More studies are needed to explore the balance point to leverage the benefit while avoiding the negative effects of short-form videos. Lastly, PAHIOWS recruited only community older women aged 60-70 years; extrapolating the findings from this study to a sample of male and female older adults or different age cohorts should be done cautiously.

### Conclusions

Our study found that short-form videos, which are being increasingly viewed by older adults, introduced both positive and negative impacts on their PA. Exposure to content related to PA would increase the minutes older adults spent on MVPA per day, while the duration of exposure would decrease their steps counts and minutes spent on LPA and MVPA. However, neither of the exposures were able to predict the achievement of spending 150 minutes or more per week on MVPA among older women.

## Abbreviations

LPA: low-intensity physical activity
MVPA: moderate-to-vigorous physical activity
OR: odds ratio
PA: physical activity
PAHIOWS: Physical Activity and Health in Older Women Study
